# Vaccination against galectin-1 promotes cytotoxic T-cell infiltration in melanoma and reduces tumor burden

**DOI:** 10.1007/s00262-021-03139-4

**Published:** 2022-01-11

**Authors:** Julia Femel, Luuk van Hooren, Melanie Herre, Jessica Cedervall, Falk Saupe, Elisabeth J. M. Huijbers, Danielle R. J. Verboogen, Matthias Reichel, Victor L. Thijssen, Arjan W. Griffioen, Lars Hellman, Anna Dimberg, Anna-Karin Olsson

**Affiliations:** 1 Department of Medical Biochemistry and Microbiology, Science for Life Laboratory, Uppsala University, Biomedical Center, Box 582, 75123 Uppsala, Sweden; 2 Department of Immunology, Genetics and Pathology, Rudbeck Laboratory, Uppsala University, 75185 Uppsala, Sweden; 3 Department of Radiation Oncology, Amsterdam UMC, Amsterdam, The Netherlands; 4 Present Address: Angiogenesis Laboratory, Department of Medical Oncology, Cancer Center Amsterdam (CCA), Amsterdam UMC, De Boelelaan 1117, 1081 HV Amsterdam, The Netherlands; 5 Department of Cell and Molecular Biology, Uppsala University, Biomedical Center, Box 596, 75124 Uppsala, Sweden; 6 Present Address: Division of Tumor Biology and Immunology, Netherlands Cancer Institute, Oncode Institute, Box 596, 1066CX Amsterdam, The Netherlands

**Keywords:** Galectin-1, Immunization, T cells, Granzyme B, Cancer vaccine

## Abstract

Galectin-1 (Gal1) is a glycan-binding protein that promotes tumor progression by several distinct mechanisms. Through direct binding to vascular endothelial growth factor (VEGF)-receptor 2, Gal1 is able to induce VEGF-like signaling, which contributes to tumor angiogenesis. Furthermore, several studies have demonstrated an immunosuppressive function of Gal1 through effects on both effector and regulatory T cells. Elevated Gal1 expression and secretion have been shown in many tumor types, and high Gal1 serum levels have been connected to poor prognosis in cancer patients. These findings suggest that therapeutic strategies directed against Gal1 would enable simultaneous targeting of angiogenesis, immune evasion and metastasis. In the current study, we have analyzed the potential of Gal1 as a cancer vaccine target. We show that it is possible to generate high anti-Gal1 antibody levels in mice immunized with a recombinant vaccine protein consisting of bacterial sequences fused to Gal1. Growth of Gal1 expressing melanomas was significantly impaired in the immunized mice compared to the control group. This was associated with improved perfusion of the tumor vasculature, as well as increased infiltration of macrophages and cytotoxic T cells (CTLs). The level of granzyme B, mainly originating from CTLs in our model, was significantly elevated in Gal1 vaccinated mice and correlated with a decrease in tumor burden. We conclude that vaccination against Gal1 is a promising pro-immunogenic approach for cancer therapy that could potentially enhance the effect of other immunotherapeutic strategies due to its ability to promote CTL influx in tumors.

## Introduction

Galectin-1 (Gal1) belongs to a conserved family of glycan-binding lectins defined by their affinity for β-galactoside, which is confined to a shared carbohydrate recognition domain (CRD) [1]. To date, fifteen mammalian galectins have been identified, consisting of either a single CRD or two linked CRDs. Most galectins are found both intra- and extracellularly, despite the lack of a classical signal peptide [2]. Because the CRDs can homodimerize or even oligomerize, galectins can increase their binding valency, which allows them to interact with multiple glycans at the same time. Extracellularly, this multivalency facilitates interactions between cells or between cells and the matrix. In addition, it allows clustering of, e.g., cell surface receptors, which enhances cell signaling. A number of cell surface and extracellular matrix glycoproteins have been shown to bind the galectin CRD, such as integrins, T cell glycoproteins, laminin, fibronectin and vitronectin [2]. It is now well established that galectins play an important role in tumor development and progression. Most notably, Gal1 has been shown to promote tumor progression at different stages and via distinct mechanisms, including immunosuppressive and pro-angiogenic effects [3]. In line with its tumor-promoting activities, Gal1 is highly expressed in many tumor types, both by the tumor cells and by the tumor endothelium [4]. Several reports point to a prominent role for Gal1 in stimulation of tumor angiogenesis. Mice deficient for Gal1 shows reduced tumor growth due to hampered tumor vascularization [5] and silencing of Gal1 in experimental models of Kaposi’s sarcoma [6] and human prostate cancer [7] reduced tumor vascularization. More recently, Gal1 was demonstrated to bind directly to vascular endothelial growth factor receptor-2 (VEGFR2) on endothelial cells via N-linked glycans [8]. This interaction induced VEGF-like signaling in the endothelium, in agreement with the previously described role of Gal1 as an endothelial growth factor [9].

In addition to its ability to induce and maintain angiogenesis, Gal1 can promote tumorigenesis via its immunosuppressive properties including induction of T-cell apoptosis [10], blocking the production of pro-inflammatory cytokines [11], inhibition of T-cell transendothelial migration [12] and by dendritic cell-induced T-cell tolerance [13]. More recently, Gal1 was shown to upregulate expression of programmed death ligand 1 (PD-L1) in tumor endothelium, thereby inducing an immune-suppressive barrier and prevention of T cell migration into the tumor [14]. Knockdown of Gal1 expression in 4T1 mouse mammary carcinoma reduced the number of immunosuppressive regulatory T cells in the tumor and tumor draining lymph nodes and resulted in a reduced metastatic burden [15]. Indeed, a pro-metastatic role for Gal1 was suggested already in 1999, based on the finding that tumor cell adhesion to endothelial cells was enhanced by Gal1 expression [16].

The findings described above suggest that targeting of Gal1 could provide several advantages as a cancer therapy, due to simultaneous suppression of tumor angiogenesis, immune escape and metastasis [17]. In agreement, several studies report that anti-Gal1 monoclonal antibody (mAb) treatment in experimental tumor models provides a therapeutic benefit without apparent side effects [6, 7, 18, 19], confirming Gal1 as a highly interesting target in tumor therapy. Monoclonal antibody-based therapies have made an important contribution to current treatment strategies for cancer, autoimmune disease and ocular disease. However, the cost for these new drugs puts a significant strain on the health-care economy, resulting in limited availability for patients. Vaccination to induce endogenous humoral immune responses against self-molecules could therefore provide a cost-efficient alternative to monoclonal antibody-based therapies for cancer and other diseases. In the current study, we address the potential of such an approach by exploring the efficacy of galectin-1 vaccination in melanoma.

## Materials and methods

### Cell culture

B16 melanoma cells (ATCC) were cultured in DMEM Glutamax (31,966,021, Invitrogen, Carlsbad, USA) containing 10% FBS (Euroclone, Pero, Italy) and 1% penicillin/streptomycin (SVA, Uppsala, Sweden) at 37 °C and 5% CO_2_. The cells were not authenticated after purchase but retained melanin-production and were regularly tested negative for mycoplasma using PCR [20] (last date for testing: March 3, 2020).

### Expression and purification of recombinant proteins

A cDNA encoding murine Gal1 (GenScript, Piscataway, NJ, the USA) was *N*-terminally fused to a His-tag (6 × His) and subcloned into the pET-21a expression vector using BamH1 and Xho1 (pET-21a-mGal1). For generation of TRX-mGal1, His-mGal1 was inserted in frame downstream of the bacterial TRX sequence in a pET-21a vector using BamH1 and Xho1 (pET-21a-TRX-mGal1) [21]. The TRX expression vector (pET-21a-TRX) was generated as described in [22].

The vectors pET-21a-TRX-mGal1, pET-21a-mGal1 and pET-21a-TRX were transformed into *Escherichia coli* (*E. coli*) Rosetta gami (DE3) (Novagen; EMD Chemicals) for expression of the fusion proteins as previously described [22–24]. Expression of mGal1 and TRX was induced at 37 °C for 4 h, while TRX-mGal1 protein expression was induced at 22 °C for 16 h to improve solubility of the protein. The fusion proteins were purified as previously described [22], using one-step purification with Ni–NTA agarose (Qiagen) and elution with imidazole (Sigma-Aldrich). Protein-containing fractions were dialyzed against PBS pH 7.4 (TRX and TRX-mGal1) or 10 mM Tris pH 9.0 (mGal1) using a Spectra/Por cellulose ester membrane (6–8 kDa MW cutoff; Spectrum Medical Industries), and final protein concentration was determined by Pierce™ BCA protein assay (Thermo Fisher Scientific). Purified fractions of TRX-mGal1, mGal1 and TRX protein were analyzed by mass spectrometry to confirm their identity. The resulting recombinant proteins and their molecular weights were: TRX-mGal1 (28 kDa), mGal1 (16 kDa) and TRX (13 kDa).

### Animal studies

Animal work was approved by the local animal ethics committee (C227/10, C114/13, C129/15) and performed according to the United Kingdom Coordinating Committee on Cancer Research (UKCCCR) guidelines for the welfare of animals in experimental neoplasia [25]. Mice were anesthetized with isoflurane (Forene; Abbott) during immunization, tumor cell injection and measurements of tumor volume. Female mice were used in this study to avoid single housing of males due to aggressive behavior.

### Immunization, blood sampling and tumor growth analysis

Eight-week-old female C57BL6/J (Taconic Biosciences) mice (*n* = 10 mice/per group) were immunized in the groin with an emulsion containing 100 µg recombinant TRX-mGal1 protein or 50 µg recombinant TRX protein (control), mixed 50:50 with the squalene-based adjuvant Montanide ISA 720 (SEPPIC, France), including 50 µg CpG oligo 1826 (Sigma-Aldrich). The mice received two booster injections into the opposite groin (booster 1 3 weeks after the first immunization, booster 2 2 weeks after booster 1). Blood samples were drawn two weeks after the second booster injection for detection of anti-Gal1 antibody levels in serum.

Mice were inoculated with 100 µl cell suspension containing 0.5 × 10^6^ B16 melanoma cells into the left flank two weeks after the second booster. One mouse in the TRX group (control) was lost during the study due to ulceration of the tumor. Mice were euthanized when tumors reached maximum allowed size in at least one individual (approximately 14 days after inoculation). Mice were terminally anesthetized by intraperitoneal injection of 2% Avertin (Sigma-Aldrich) and retro-orbitally injected with 150 μl FITC-coupled Lycopersicon esculentum lectin solution (FITC-LEL, 0.5 mg/ml; Vector Laboratories). Subsequently, mice were perfused with 10 ml PBS followed by 10 ml of 2% paraformaldehyde (PFA) through the heart. Tumors were dissected, weighed and cryopreserved in 30% sucrose overnight at 4 °C. The tissue was then snap-frozen in isopentane/dry-ice and stored at −70 °C until use.

### Western Blot

Conditioned medium (c.m.) from B16 cells cultured for 48 h was collected before harvesting the cells in lysis buffer (0.5% Triton X-100, 0.5% sodium-deoxycholate, 20 mM Tris–HCl pH 7.5, 10 mM EDTA, 150 mM NaCl) including protease inhibitors (Halt Protease Inhibitor Cocktail #87,786, Thermo Scientific). Cell lysate was obtained by freeze–thaw cycles and vortexing with glass beads. The protein concentration was determined with BCA protein assay (#23,223, Thermo Scientific). Protein from conditioned medium (c.m.) was precipitated using methanol to increase protein yield and visualization in Western blot. Conditioned medium was mixed with ice-cold methanol (1:10 dilution) and stored at −20 °C for 24 h. The mixture was centrifuged at 4500x*g* for 30 min to collect all precipitated proteins. Cell lysate (25 µg) and precipitated c.m. from the same cells were separated using 4–12% Bis–Tris gels and the Novex NuPAGE system (Life Technologies). For comparison, 1/12 of the total lysate and 1/10 of the c.m. were loaded on the gel. After protein transfer, the membrane was blocked with Odyssey blocking buffer (LI-COR Biosciences) and incubated with rabbit anti-human Gal1 antibody (1:2000; 500-P201, Peprotech, cross-reactive with mouse Gal1) overnight at 4 °C. The membrane was subsequently incubated with anti-rabbit IRDye CW 800 nm (1:10 000; 926–32,213, LI-COR Biosciences). The membrane was scanned using the Odyssey Infrared Imaging system and analyzed with Software Image Studio Lite (LI-COR Biosciences).

### ELISA

ELISA plates (Multiwell Immuno Plate, Maxisorp, 96 well; M9410, Thermo Scientific) were coated with 5 μg/ml recombinant mGal1 in PBS (pH 7.4) and blocked with horse serum. Mouse sera were diluted in 10% *E. coli* Rosetta gami (DE3) whole-cell extract (to reduce background) to a final dilution of 1:500. Anti-Gal1 antibodies were detected with 3 μg/ml biotinylated goat anti-mouse IgG (H + L) (BA-9200; Vector Laboratories) and 2 μg/ml streptavidin–horseradish peroxidase (SA-HRP, SA-5004; Vector Laboratories). All incubations were performed at 37 °C. HRP activity was detected with TMB substrate (T8665, Sigma-Aldrich), and absorbance was measured at 650 nm. All samples and blanks were assayed as duplicates.

### Sandwich ELISA for detection of Gal1 serum levels

ELISA plates (Multiwell Immuno Plate, Maxisorp, 96 well; M9410, Thermo Scientific) were coated with 8 μg/ml goat anti-mouse Gal1 capture antibody (AF1245, R&D Systems) in PBS pH 7.4 and blocked with horse serum. Subsequently, the standard (recombinant mouse Gal1; 1245-GA, R&D) or mouse sera (diluted 1:15 in PBS) were added. Rabbit anti-human Gal1 antibody (0.75 μg/ml; 500-P210, Peprotech) was used as detection antibody, followed by 3 μg/ml biotinylated goat anti-rabbit IgG (BA-1000, Vector Laboratories) and 1 μg/ml streptavidin–horseradish peroxidase. Enzymatic detection was performed as described above. A standard curve ranging from 2.5 to 80 ng/ml of recombinant mouse Gal1 was used to calculate Gal1 concentrations in the samples. Four-parameter logistics regression was used for fitting the standard curve.

### Immunofluorescence and quantification

Cryosections (5 µm) of B16 tumors from untreated mice were stained for Gal1 using a polyclonal rabbit anti-human Gal1 antibody (1:250; 500-P201, Peprotech) and co-stained for blood vessels using a rat anti-mouse CD31 antibody (1:1000; 553,370, BD Pharmingen). For analysis of various cell types and markers cryosections (5 µm) of B16 melanomas from immunized mice (TRX-Gal1 or TRX) were stained with the following antibodies: monoclonal rat anti-mouse CD31 (553,370, BD Pharmingen), monoclonal rat anti-mouse CD45 (1:200; 553,076, BD Pharmingen), monoclonal rat anti-CD68 (1:300; MCA1957; AbD Serotec), monoclonal APC-conjugated rat-anti-CD68 monoclonal (1:300; 130–102-585, Miltenyi Biotec), rat anti-mouse CD206 (1:200; MCA2235EL, BioRad), monoclonal hamster anti-CD11c (1:100; ab33483, Abcam), monoclonal rat anti-CD3 (1:200; 555,273, BD Biosciences), monoclonal rabbit anti-cleaved Caspase-3 (1:400; 9664, Cell Signaling Technology), mouse monoclonal anti-mouse/human granzyme B (1:500, clone GB11, Biolegend), monoclonal rat anti-NKp46 (1:100; 137,602, Biolegend) and monoclonal rat anti-mouse CD8 (1:200, clone 53–6.7, Biolegend). Cryosections were fixed in 100% ice-cold acetone or methanol (Gal1 and CD68 only) and blocked with 3–5% BSA/PBS, 5% BSA/PBS containing 5% horse serum (CD68 only) or 5% horse serum (CD3 only). For CD11c, staining sections were fixed in 4% PFA, and for NKp46, staining sections were not fixed or blocked.

Secondary antibodies used were: donkey anti-rat Alexa488, donkey anti-rabbit Alexa488, goat anti-rabbit AlexaFluor568, goat anti-rat AlexaFluor568, goat anti-rabbit AlexaFluor594 (all Invitrogen), goat anti-hamster Alexa647 (127-605-160, Jackson ImmunoResearch) and donkey anti-ratCy3 (712-165-150, Jackson ImmunoReserach), all diluted 1:1000. For staining of CD45, secondary antibody was diluted in the presence of 5% goat serum, and for staining of CD3, secondary antibody was diluted in the presence of 5% goat serum and 2% mouse serum. Nuclei were visualized with Hoechst 33,342 (1 µg/ml, VWR International), and sections were mounted with Fluoromount-G (0100-01, Southern Biotech). The FITC signal after FITC-LEL perfusion was enhanced with an anti-FITC antibody (1:500; 71-1900; Life Technologies).

For quantifications, one section per tumor was stained and analyzed for each marker. Four to 12 images of each tumor section, depending on the tumor size, were taken at random in peripheral and inner tumor areas with a Nikon Eclipse 90i microscope (with a DS-Qi1Mc monochrome CCD camera and Nikon NIS Elements AR 3.2 software), using the 20× objective (Plan Apo 0.75) or the 10X objective (Plan Apo 0.45) at the same exposure time for each marker, respectively. ImageJ64 10.2 software (National Institutes of Health, the USA) was used for quantification of areas (area % of 20× or 10× field). Quantification of number of cells staining positive for the respective marker(s) was counted per 20× field, and the average was calculated for each individual. To quantify the numbers of M1 and M2 macrophages, cells double-positive for CD68 and CD11c (M1) or CD68 and CD206 (M2) were divided with the total number of CD68 positive cells.

### Statistical analysis

Statistical analyses were performed using GraphPad Prism 5.0c. Statistical analyses in this study were performed using Student’s *t* test, or the non-parametric two-tailed Mann–Whitney test for data not following normal distribution. Normality was tested with the Shapiro–Wilk test and D'Agostino and Pearson omnibus test. Correlation was determined with Pearson correlation. * is defined as *p* ≤ 0.05, *** as *p* ≤ 0.0001.

## Results

### Generation of a Gal1 vaccine

**Fig. 1:**
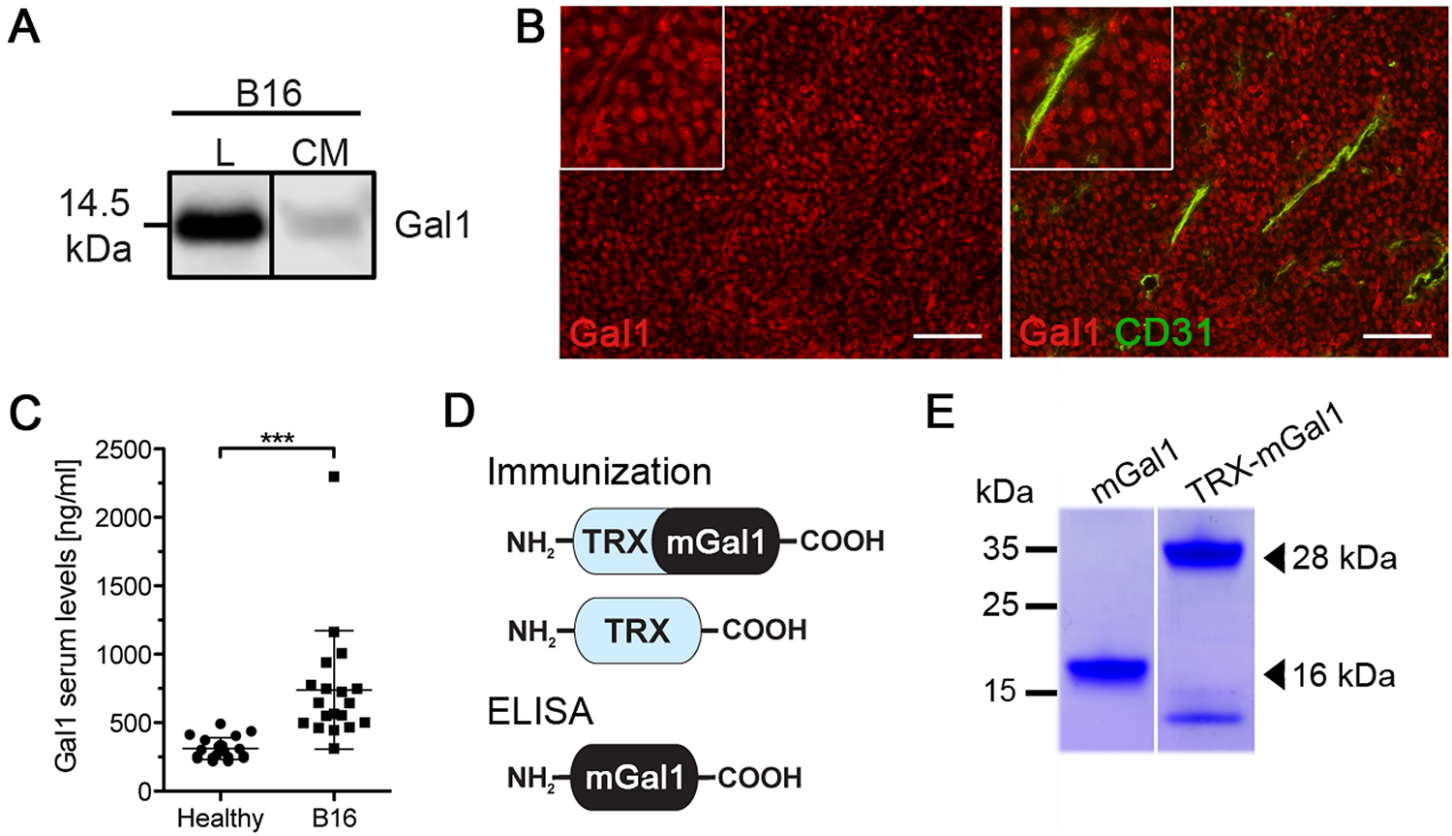
Generation of a Gal1 vaccine for experimental melanoma. **a** Western blot for Gal1 in cell lysate (L) and conditioned media (CM; methanol-precipitated) from cultured B16 melanoma cells. **b** Immunostaining for Gal1 (red) and blood vessels (CD31, green; right panel) in tumors derived from B16 melanoma cells. Scale bar: 100 µm. **c** Gal1 measured in serum from healthy and tumor-bearing mice using a sandwich ELISA. Each dot represents an individual. The difference in Gal1 concentration between the groups was assessed by Mann–Whitney *U* test (data not normally distributed), *p* < 0.0001. **d** Schematic illustration of the recombinant proteins used for immunization of mice (TRX-mGal1 and TRX) and detection of anti-Gal1 antibodies in ELISA (mGal1). **e** Purified proteins separated by SDS-PAGE, showing His-tagged mGal-1 (lane 1) at 16 kDa and TRX-mGal1 (lane 2) at 28 kDa

To address the potential tumoristatic effect of a Gal1 vaccine, we selected B16 melanoma as a model. Both human and murine melanomas have previously been shown to express and secrete high levels of Gal1 [26]. Moreover, Gal1 has been demonstrated to possess pro-angiogenic [9] as well as immunosuppressive functions [26] in B16 murine melanoma. Firstly, we confirmed the expression and secretion of Gal1 by B16 melanoma cells using Western blot of cell lysate and conditioned media from cultured B16 melanoma cells (Fig. 1a). Secondly, expression of Gal1 was observed by immunostaining of B16 tumor tissue, showing expression in both tumor cells and the tumor endothelium (Fig. 1b). Finally, the Gal1 concentration was determined in serum samples collected before and 14 days after tumor inoculation, using a sandwich ELISA. There was a significant elevation of Gal1 serum levels after tumor cell grafting (Fig. 1c). Collectively, these data identify the B16 melanoma model as a suitable model to explore the efficacy of Gal1 vaccination. For this, we designed, expressed and purified a recombinant Gal1 vaccine protein consisting of bacterial thioredoxin (TRX; a foreign domain) fused to mouse Gal1 (mGal1; the endogenous target), generating the TRX–mGal1 fusion protein vaccine. In addition, recombinant mouse Gal1 without the TRX-domain was produced (mGal1), with the purpose to be used as an antigen in ELISA measuring anti-Gal1 antibody levels in vaccinated mice (Fig. 1d). Recombinant TRX [22] was used for immunization of control mice. All recombinant vaccine proteins were generated with a His-tag to enable one-step purification. As predicted, purified TRX-mGal1 and mGal1 proteins migrated as 28 and 16 kDa, respectively (Fig. 1e). The lower band in the TRX-mGal1 lane represents a partial degradation product of the TRX-mGal1 protein including the His-tag.

### Immunization against Gal1 suppresses tumor growth

**Fig. 2:**
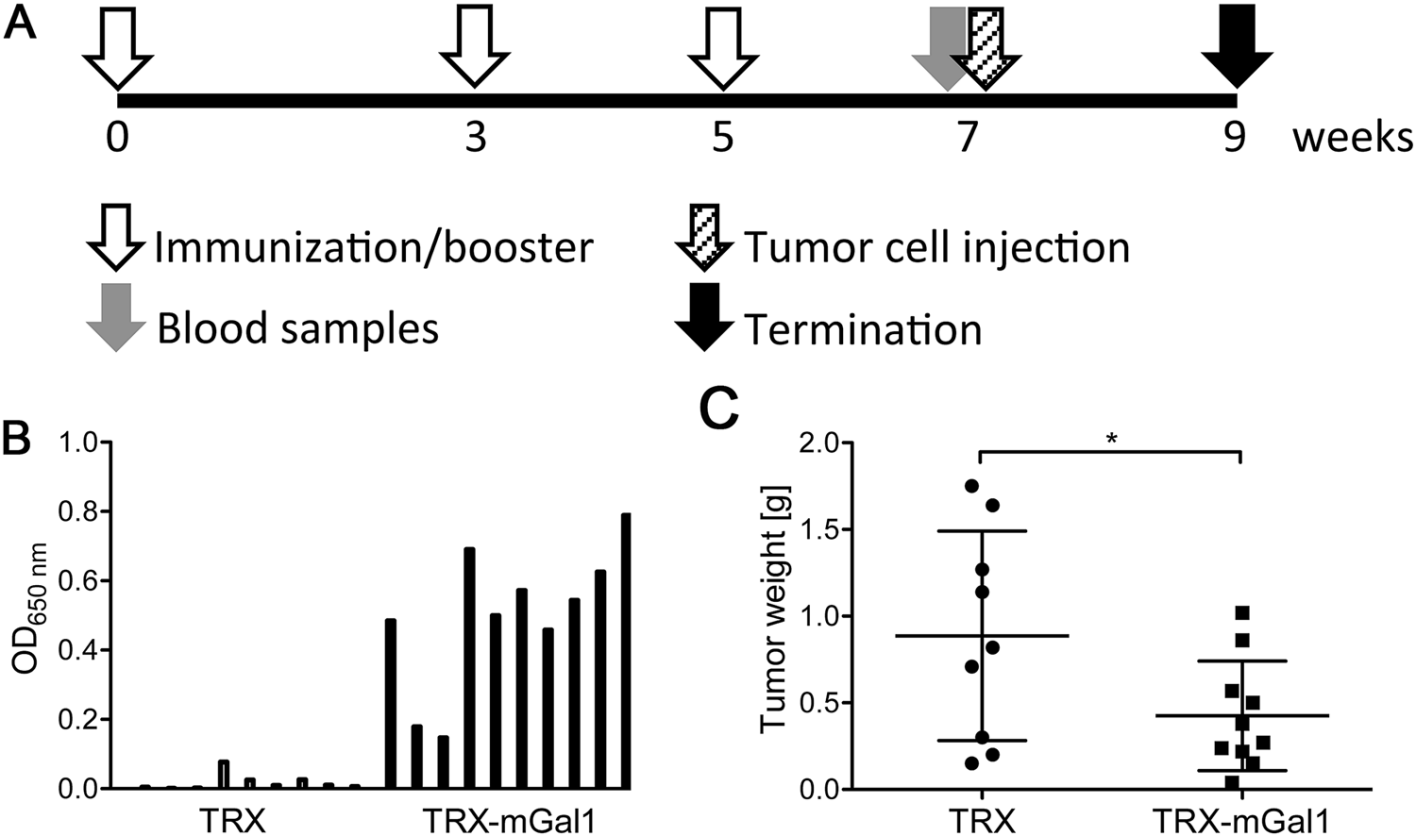
Vaccination against Gal1 generates anti-Gal1 antibodies and suppresses tumor growth. **a** Immunization schedule for C57/BL6 mice with TRX (control) or TRX-mGal1 protein; time points for blood sampling, tumor challenge and termination are indicated. **b** Anti-Gal1 antibody levels measured by ELISA in serum from TRX and TRX-mGal1 vaccinated mice two weeks after the second booster immunization and before tumor inoculation. Bars represent individual animals. **c** Tumor weights on the day of termination in mice immunized with TRX (control) or TRX-mGal1. The graph illustrates mean values ± SD, and each dot represents an individual. The difference in tumor weight between the groups was assessed by Student’s *t* test, *p* = 0.0489

C57BL/6 J mice (*n* = 10/group) were immunized with the TRX-mGal1 fusion protein or TRX alone (control), together with the adjuvant Montanide ISA 720 (M720)/CpG [22]. After primary vaccination, the mice received two booster injections of the vaccine before tumor cell injection to ensure an efficient immunization (see experimental schedule Fig. 2a). Serum sampling was done two weeks after the second booster immunization. Anti-Gal1 antibody levels were measured by ELISA using recombinant mGal1 as antigen. All TRX–mGal1 vaccinated mice showed a clear anti-Gal1 immunoreactivity in their serum (Fig. 2b). Vaccinated mice were inoculated subcutaneously on their left flank with 0.5 million B16 cells, and tumor growth was allowed to proceed until day 14 after cell grafting. The average tumor weight in the Gal1 immunized group was significantly smaller at day 14 compared to the TRX immunized group (Fig. 2c).

### Tumor vascular perfusion is improved after vaccination against Gal1

**Fig. 3:**
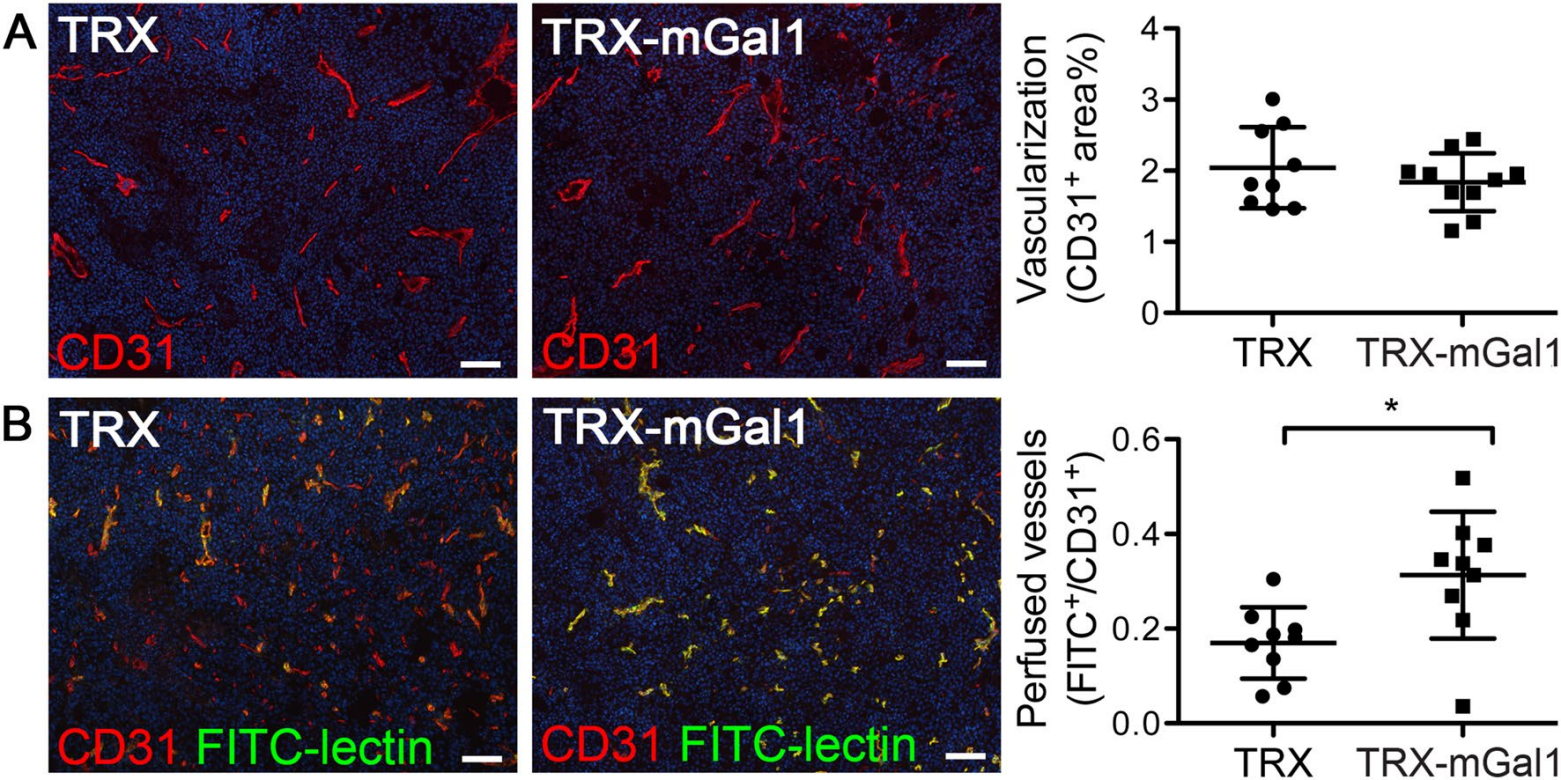
Tumor vascular perfusion is improved after vaccination against Gal1. **a** Representative immunostaining for blood vessels (CD31, red) and quantification of CD31-positive area (% of 10× field). B) FITC-LEL perfused blood vessels (green) and immunostaining for blood vessels (CD31, red). The proportion of FITC-LEL positive blood vessels was determined (FITC^+^ area/CD31^+^ area). The graphs illustrate mean values ± SD, and each dot represents an individual. Statistical differences between the groups were determined by Student’s t test (CD31^+^ cells, *p* = 0.3754 and FITC^+^ area/CD31^+^ area, *p* = 0.0131). Scale bar: 100 µm

Based on the well-documented pro-angiogenic effect of Gal1, we analyzed the extent of vascularization in tumors from TRX and TRX-mGal1 vaccinated mice by immunostaining for CD31. Quantification of the CD31-positive area did not reveal any change in tumor vascularization in the Gal1 vaccinated group, compared to the control group (Fig. 3a). Targeting of Gal1 with a monoclonal antibody was reported to promote normalization of tumor vessels [8], an effect that would not be detected by CD31 staining alone. Normalization of the tumor vasculature is characterized by improved perfusion of blood vessels. To determine whether Gal1 immunization affected vascular perfusion of the tumors, we injected mice intravenously with FITC-coupled Lycopersicon esculentum lectin (FITC-LEL). FITC-LEL binds to the lumen of blood vessels and can therefore be used to analyze the proportion of perfused vessels. Indeed, vascular perfusion was significantly improved in tumors from Gal1 immunized mice (Fig. 3b). Thus, while the vaccination did not appear to reduce tumor vascularization, it did result in vascular normalization.

### Enhanced leukocyte recruitment to tumors in anti-Gal1 immunized mice

**Fig. 4:**
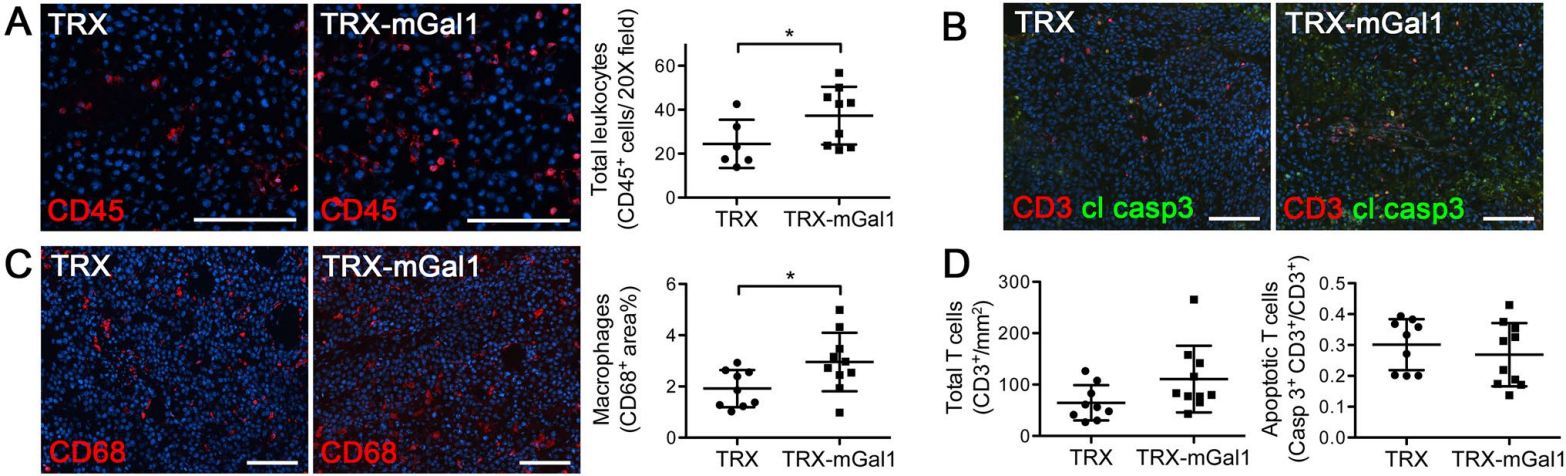
Leukocyte recruitment to tumors is enhanced in Gal1 immunized mice. **a** Representative immunostaining for leukocytes (CD45, red) and quantification of CD45^+^ cells (per 20× field). The difference in CD45^+^ cell infiltration between the groups was assessed by Mann–Whitney U test (data not normally distributed), *p* = 0.0496. B) Representative immunostaining for T cells (CD3, red) and apoptotic T cells by cleaved caspase-3 staining (green). C) Representative immunostaining for macrophages (CD68, red) and quantification of CD68-positive area (% per 10X field). The difference in macrophage infiltration was determined by Student’s *t* test, *p* = 0.0324. D) Quantification of CD3^+^ cells per mm^2^ (left) and proportion of apoptotic CD3^+^ cells (right). Statistical differences between the groups were determined by Mann–Whitney *U* test (data not normally distributed; CD3^+^ cells, *p* = 0.0535 and CD3^+^/cleaved caspase-3^+^ double-positive cells, *p* = 0.3154). All graphs illustrate mean values ± SD, and each dot represents an individual. Scale bars: 100 µm

Normalization of the tumor vasculature is commonly associated with elevated endothelial activation and increased infiltration of immune cells into the tumor [27–29]. To address if this was also the case in tumors from Gal1 vaccinated mice, we performed immunohistochemical staining for the pan-leukocyte marker CD45. As illustrated in Fig. 4a, there was a significant increase in tumor-infiltrating CD45-positive leukocytes in Gal1 immunized mice. To further elucidate which cell types that constitute the increased amount of leukocytes, we immunostained the tumor tissues for macrophages (CD68) and T cells (CD3). While there was a trend to an increased number of CD3^+^ T cells in the TRX-mGal1 group (Fig. 4b and d, left panel, *p* = 0.0535), the number of macrophages was significantly increased in B16 tumors from Gal1 immunized mice compared to TRX immunized mice (Fig. 4c). Since Gal1 has been demonstrated to induce T cell apoptosis [10], we analyzed whether the trend toward more CD3^+^ T cells in the tumor was paralleled by reduced apoptosis in this cell population in Gal1 vaccinated mice. We could, however, not detect any changes in the number of apoptotic (cleaved caspase-3^+^) CD3^+^ T cells in Gal1 vaccinated mice (Fig. 4b and d, right panel).

**Fig. 5:**
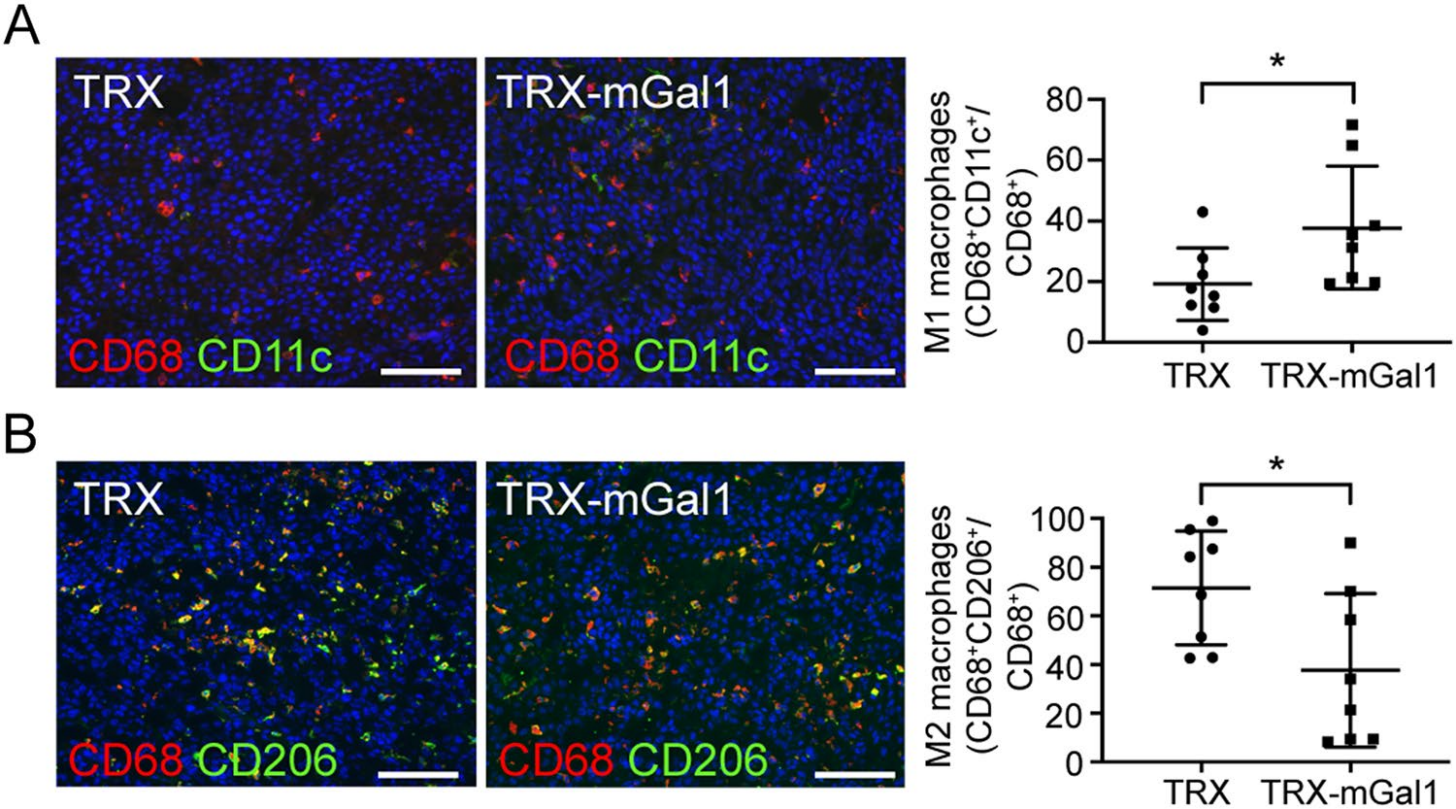
Gal1 immunized mice have more M1 and less M2 macrophages compared to control immunized mice. **a** Representative immunostaining (CD68 and CD11c) and quantification of M1 macrophages in tumors from TRX (control) and TRX-mGal1 immunized mice (*p* = 0.0379). **b** Representative immunostaining (CD68 and CD206) and quantification of M2 macrophages in tumors from TRX (control) and TRX-mGal1 immunized mice (*p* = 0.0477). For quantification, double-positive cells (CD68 + CD11c + for M1 and CD68 + CD206 + for M2) were counted and divided by the total number of CD68 + cells in the same area. Statistical differences between the groups were determined by Mann–Whitney *U* test (data not normally distributed). All graphs illustrate mean values ± SD, and each dot represents an individual. Scale bars: 100 µm

Gal1 has been demonstrated to shape the immune landscape in tumors in several ways, for example, by polarization of macrophages toward a tumor-promoting (M2) phenotype. To investigate if the macrophage phenotype was affected by Gal1 vaccination, we performed immunostaining for the M1 marker CD11c and the M2 marker CD206 together with CD68. As shown in Fig. 5, the proportion of CD11c + CD68 + macrophages increased (Fig. 5a), while the proportion of CD206 + CD68 + macrophages decreased (Fig. 5b).

### Cytotoxic activity of CD8^+^ T cells is increased in tumors from Gal1 immunized mice

**Fig. 6:**
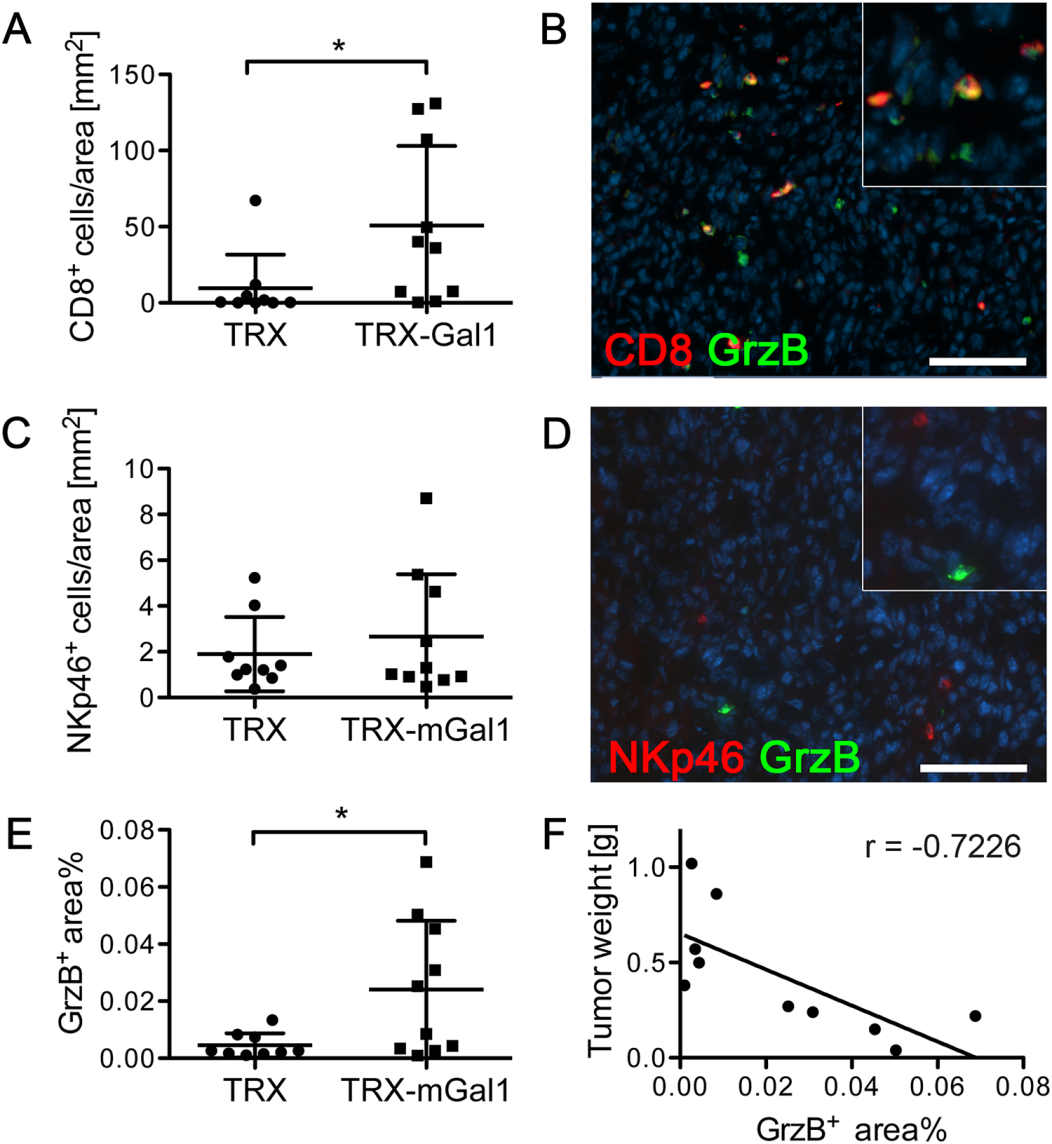
Cytotoxic activity of CD8^+^ T cells is increased in tumors from Gal1 immunized mice. **a** Quantification of CD8^+^ cells per mm^2^ in tumors from TRX (control) or TRX-mGal1 immunized mice (*p* = 0.0247). **b** Representative immunostaining for cytotoxic T cells (CD8, red) and granzyme B (GrzB, green). **c** Quantification of NKp46-positive cells per mm^2^ (*p* = 0.9048). **d** Representative immunostaining of NK cells (NKp46, red) and granzyme B (GrzB, green). **e** Quantification of granzyme B (positive area % per 10X field; *p* = 0.0373). **f** Correlation of GrzB-positive area (% of 10× field) and tumor weight in TRX-mGal1 immunized mice at termination of the experiment (*p* = 0.0182, Pearson correlation coefficient *r* = -0.7226). Statistical differences between the groups were determined by Mann–Whitney *U* test (data not normally distributed). All graphs illustrate mean values ± SD, and each dot represents an individual. Scale bars: 100 µm

While we did not find a significant difference in the total number of CD3^+^ T cells, we examined whether the number of CD8^+^ cytotoxic T cells (CTLs) in the tumors was altered by vaccination against Gal1. Immunohistochemical staining for CD8 revealed a significant increase of CD8^+^ CTLs in tumors from Gal1 vaccinated mice (Fig. 6a and b). Immunohistochemical staining for NKp46^+^ natural killer (NK) cells, another cytotoxic leukocyte with capacity to recognize and kill tumor cells, did not reveal any difference between the two groups of mice (Fig. 6c and d). In general, NK cells were also less numerous than the CD8^+^ CTLs. Immunohistochemical staining for granzyme B (GrzB), the main mediator of CTL activity, showed significantly higher levels of this serine protease in tumors from Gal1 immunized mice compared to the control group (Fig. 6e). Moreover, the GrzB immunostaining largely co-localized with the CD8^+^ T cell population (Fig. 6b), although a few GrzB + CD8-cells were seen. Of note, NK cells have also been reported to express GrzB. However, co-staining for NK cells and GrzB showed that GrzB-positive cells were largely NKp46-negative (Fig. 6d), indicating that GrzB is mainly produced by CD8^+^ cytotoxic T cells in our model. Interestingly, there was a strong negative correlation between the amount of GrzB present in a tumor and the corresponding tumor weight in the Gal1 immunized group (Fig. 6f). These data suggest that the reduction in tumor volume in the Gal1 vaccinated group was mediated by an increase in CTL-derived GrzB activity.

## Discussion

Gal1 has emerged as a pleiotrophic immunosuppressive molecule in the tumor microenvironment that differentially regulates both innate and adaptive components of anti-tumor immunity [30]. Moreover, Gal1 promotes angiogenic responses, especially in hypoxic and anti-VEGF refractory tumors [3, 8]. Collectively, these findings render Gal1 a highly interesting target in cancer therapy. In the current study, we evaluated the therapeutic potential of a Gal1-targeting vaccine in an experimental melanoma model in vivo. We show that it is possible to circumvent self-tolerance and induce a strong antibody response against Gal1 by vaccination, without any apparent side effects. Importantly, tumor burden was significantly reduced in mice vaccinated against Gal1.

The vaccination strategy employed in the current study is based on the use of a recombinant fusion protein consisting of a self (the target) and a non-self-domain. We have carefully elucidated the mechanism and requirements behind immunization using this method [21, 22, 24, 31] in several previous studies, and we have shown that the immune response is mediated by generation of antibodies against the target molecule. The increased infiltration of CD8^+^ T cells in tumors from Gal1-vaccinated mice is therefore an effect resulting from antibody-mediated neutralization of Gal1 expressed and secreted by the tumor.

The pro-angiogenic effects of Gal1 are well documented [5–9]. Excessive angiogenic stimulation, as in tumors, will suppress expression of leukocyte adhesion molecules such as ICAM-1 on the endothelium [27–29]. Consequently, disruption of this signal by anti-angiogenic therapy (in the shape of antibodies or kinase inhibitors) has been demonstrated to normalize expression of vascular adhesion molecules and thereby facilitate leukocyte adhesion, transmigration and infiltration in tumor tissue [27–29]. Similarly, we found that neutralization of Gal1 by vaccination improved tumor vascular perfusion and increased infiltration of CD45^+^ leukocytes into the tumors, indicating a functional normalization of the tumor vasculature.

Among the CD45^+^ leukocytes, there was a significant increase in the CD68^+^ macrophage and the CD8^+^ T cell population. Although there are still limited data on the role of Gal1 in macrophage recruitment and differentiation, Gal1 has previously been reported to affect myeloid cell accumulation within tumors. In the orthotopic GL261 mouse glioma model, lentiviral knockdown of Gal1 in the tumor cells significantly decreased the amount of brain-infiltrating F4/80^+^ macrophages and myeloid-derived suppressor cells (MDSCs) [32]. This is in contrast to our data showing an elevated number of CD68^+^ macrophages in B16 melanomas after Gal1 vaccination. Potential explanations for these differences could be that (1) the F4/80^+^ and CD68^+^ macrophages represent distinct populations that are regulated differently by Gal1, (2) glioma and melanoma respond in distinct ways to reduced levels of Gal1 or that (3) neutralization of circulating Gal1 by antibodies generates a different response in the tumor microenvironment compared to knockdown of Gal1 in the tumor cells. At least in the B16 melanoma model used in this study, F4/80 and CD68 identify largely overlapping populations of macrophages. There are slightly more CD68 + cells than F4/80 + cells, but all F4/80 + cells are CD68 + in the B16 tumors. Therefore, we believe that CD68 is a suitable macrophage marker in this model. Furthermore, when analyzing the macrophage phenotype in the Gal1 immunized mice, we found that a higher proportion of the CD68 + macrophages expressed the M1 marker CD11c + , while the proportion that were CD206 + (M2 phenotype) was reduced. These findings are in agreement with previous reports showing that Gal1 can induce polarization of macrophages toward an M2 phenotype [30], highlighting an additional advantage of neutralizing Gal1 in individuals with cancer.

An elevated number of tumor-infiltrating CD8^+^ T cells after Gal1 vaccination is in good agreement with previous studies showing that Gal1 can inhibit adhesion [33] and transendothelial migration of T cells [12]. Furthermore, treatment of tumor-bearing mice with a monoclonal anti-Gal1 antibody led to normalization of the tumor vasculature in Lewis lung carcinoma (LLC) and B16 melanomas and an elevation of the number of CD8^+^ T cells in the tumor microenvironment [6–8, 18]. The increase in tumor-infiltrating CTLs in tumors from Gal1 vaccinated mice was paralleled by an elevated level of GrzB. There was also a significant correlation between high GrzB expression and reduced tumor burden, indicating that elevated CTL activity is responsible for the tumoristatic effect of the Gal1 vaccine. More GrzB in tumors from Gal1 vaccinated mice could simply reflect the higher number of CTLs in the tumors. However, it is possible that neutralization of Gal1 by the vaccine also promotes activation of the CD8^+^ T cells, since Gal1 has been shown to suppress survival and activation of CTLs [26]. In agreement, Nambiar et al. have shown that Gal1 induces an upregulation of the immunosuppressive PD-L1 on the tumor endothelium, thus transforming it into an immune-suppressive barrier [14]. This study highlights the potential benefit of combining immune-checkpoint inhibitor therapy with a Gal1 targeting strategy.

Another cytotoxic immune cell that has been implicated as a target for the immunosuppressive actions of Gal1 is the NK cell. Knockdown of Gal1 in the murine GL261 glioma model resulted in almost complete eradication of the tumors. This effect was attributed to enhanced cytotoxic activity of NK cells, including increased expression of GrzB in mice on a RAG1-/- background, which lack cells of the adaptive immune system [34]. We also investigated whether the anti-tumor effects in Gal1 vaccinated mice were mediated by NK cells. However, recombinant Gal1 did not affect NK cell activation in vitro in our hands (data not shown). In addition, cells positive for the NK cell marker NKp46 were largely negative for GrzB, suggesting that CD8 + T cells were the major source of GrzB in this melanoma model. We did note a few GrzB + cells that did not appear to be either CTLs or NK cells. We can only speculate which cell type they represent, but GrzB expression has been reported also in other types of hematopoietic cells, for example, B cells. It has been reported that B cells can secrete GrzB without simultaneous expression of perforin, needed for cellular cytotoxicity, and that this B cell-derived GrzB expression plays a role in cancer immunosurveillance [35]. If Gal1 can influence GrzB expression in B cells remains to be investigated.

Altogether, the current study shows that it is feasible to develop an efficient therapeutic vaccine targeting Gal1. Neutralization of tumor-derived Gal1 promoted normalization of the tumor vasculature, increased CTL infiltration and cytotoxic activity in tumors and reduced tumor burden. These biological effects are in good agreement with previous studies using either knockdown of Gal1 in tumor cells or administration of monoclonal antibodies (mAbs) targeting Gal1. In comparison to tumor cell-specific knockdown of Gal in cancer patients, vaccination to generate an antibody response to Gal1 is a significantly more realistic approach. Although administration of mAbs as therapeutic agents is feasible and in clinical use for cancer and autoimmune disease, the high cost associated with these drugs puts a significant strain on the health care budget, and also on patient economy. As an example, treatment with the anti-VEGF antibody bevacizumab (Avastin) can cost up to 9000 USD per month. If patients are required to pay even a small part of this cost, drugs quickly become unaffordable [36]. Moreover, there is an ongoing discussion on how to take these costs into consideration when designing clinical trials. This is in contrast to the idea that clinical trials should be selected only on clinical grounds [36, 37]. Based on these incentives, it is highly relevant to investigate the potential of alternative therapies that offer equal clinical benefit, but at a lower cost. Therapeutic vaccination, defined as induction of an endogenous polyclonal antibody response, could provide such an alternative. While mAb therapy requires a few hundred milligrams of high-quality GMP (Good Manufacturing Practice)-produced recombinant antibody every second week, up to gram amounts per week, vaccines usually involve a 10,000–20,000 times lower amount of recombinant protein, which is significantly more cost effective.

During all drug development phases, safety issues are essential, and vaccines are no exception. Importantly, no adverse effects of the Gal1 vaccination were observed in the current study. While being expressed in other cells and tissues, overexpression has been reported in many different tumor cell types, both by cell surface expression and by secretion [4]. We have identified the overexpression of Gal1 in the tumor vasculature and its role in the process of angiogenesis [5]. It is this overexpression that provides the therapeutic window that can be exploited for treatment of cancer. This was initially reported to have significant therapeutic potential by the development of the Gal1 targeting peptide anginex [38] and the small molecule topomimetics [39]. Also targeting Gal1 by a specific monoclonal antibody had significant anti-tumor efficacy [6–8, 18, 19], while no adverse effects were reported. Gal1 expression in the tumor vasculature serves the tumor in three important ways: (i) It provides the tumor with increased angiogenesis, (ii) it provides the tumor with metastatic potential, and (iii) it increases the barrier function of the tumor vasculature [40] for the formation of a leukocyte infiltrate, by providing an apoptotic signal to transmigrating leukocytes [10]. Off-target effects are not expected, considering the high specificity of the Gal1 targeting antibodies. However, more long-term exposure to the neutralizing anti-Gal1 antibodies is needed, preferentially in several species, to make firm conclusions. A relevant note in this context is that reduced levels of circulating Gal1, as well as increased levels of anti-Gal1 auto-antibodies, have been connected to recurrent pregnancy loss [41]. Moreover, blocking of Gal1 activity has been shown to induce a preeclampsia-like syndrome in mice [42], indicating that pregnant women may not be a suitable patient group to receive anti-Gal1 therapy.

In contrast to vaccination against infectious agents like bacteria or virus, immunization against a self-antigen is not life-long, since the immune system has mechanisms that counteract auto-immunity under normal conditions. In agreement with this, we have previously shown that the immune response induced against several self-antigens using the same fusion protein technique is reversible [22, 43], which is an important safety aspect of our approach.

Taken together, our data strongly suggest vaccination against Gal1 as a novel, potent and cost-efficient treatment strategy for cancer, which should be further explored.
