# Correction to: Vaccination against galectin-1 promotes cytotoxic T-cell infiltration in melanoma and reduces tumor burden

**DOI:** 10.1007/s00262-022-03175-8

**Published:** 2022-02-28

**Authors:** Julia Femel, Luuk van Hooren, Melanie Herre, Jessica Cedervall, Falk Saupe, Elisabeth J. M. Huijbers, Danielle R. J. Verboogen, Matthias Reichel, Victor L. Thijssen, Arjan W. Griffioen, Lars Hellman, Anna Dimberg, Anna-Karin Olsson

**Affiliations:** 1 Department of Medical Biochemistry and Microbiology, Science for Life Laboratory, Uppsala University, Biomedical Center, Box 582, 75123 Uppsala, Sweden; 2 Department of Immunology, Genetics and Pathology, Rudbeck Laboratory, Uppsala University, 75185 Uppsala, Sweden; 3 Department of Radiation Oncology, Amsterdam UMC, Amsterdam, The Netherlands; 4 Present Address: Angiogenesis Laboratory, Department of Medical Oncology, Cancer Center Amsterdam (CCA), Amsterdam UMC, De Boelelaan 1117, 1081 HV Amsterdam, The Netherlands; 5 Department of Cell and Molecular Biology, Uppsala University, Biomedical Center, Box 596, 75124 Uppsala, Sweden; 6 Present Address: Division of Tumor Biology and Immunology, Netherlands Cancer Institute, Oncode Institute, Box 596, 1066CX Amsterdam, The Netherlands

## Correction to: Cancer Immunology, Immunotherapy https://doi.org/10.1007/s00262-021-03139-4

The article Vaccination against galectin-1 promotes cytotoxic T-cell infltration in melanoma and reduces tumor burden, written by Julia Femel, Luuk van Hooren, Melanie Herre, Jessica Cedervall, Falk Saupe, Elisabeth J. M. Huijbers, Danielle R. J. Verboogen, Matthias Reichel, Victor L. Thijssen, Arjan W. Grifoen, Lars Hellman, Anna Dimberg and Anna-Karin Olsson, was originally published electronically on the publisher’s internet portal on 11 January 2021 without open access. With the author(s)’ decision to opt for Open Choice the copyright of the article changed on 08 February 2022 to © The Author(s) 2022 and this article is licensed under a Creative Commons Attribution 4.0 International License, which permits use, sharing, adaptation, distribution and reproduction in any medium or format, as long as you give appropriate credit to the original author(s) and the source, provide a link to the Creative Commons licence, and indicate if changes were made. The images or other third party material in this article are included in the article's Creative Commons licence, unless indicated otherwise in a credit line to the material. If material is not included in the article's Creative Commons licence and your intended use is not permitted by statutory regulation or exceeds the permitted use, you will need to obtain permission directly from the copyright holder. To view a copy of this licence, visit http://creativecommons.org/licenses/by/4.0/.

The original article has been corrected.

